# Potential Mechanisms of Acupuncture for Functional Dyspepsia Based on Pathophysiology

**DOI:** 10.3389/fnins.2021.781215

**Published:** 2022-01-25

**Authors:** Na-Na Yang, Chun-Xia Tan, Lu-Lu Lin, Xin-Tong Su, Yue-Jie Li, Ling-Yu Qi, Yu Wang, Jing-Wen Yang, Cun-Zhi Liu

**Affiliations:** International Acupuncture and Moxibustion Innovation Institute, School of Acupuncture, Moxibustion and Tunia, Beijing University of Chinese Medicine, Beijing, China

**Keywords:** functional dyspepsia, acupuncture, low-grade inflammation, brain-gut axis, acid exposure

## Abstract

Functional dyspepsia (FD), a common disorder of gastrointestinal function, originated from the gastroduodenum. Although the therapeutic effect of acupuncture has been investigated by various high-quality randomized controlled trials, the potential mechanisms showed obvious heterogeneity. This review summarized the potential mechanisms of acupuncture on FD in order to guide for future laboratory and clinical studies. Here, we argued that the primary cause of FD was gastroduodenal low-grade inflammation and acid exposure, which impaired mucosal integrity, caused brain-gut axis dysfunction, and impaired brain network connectivity, all of which generated various symptom patterns. Overall the clinical studies indicated that acupuncture was a promising treatment to alleviate symptoms in FD patients, whose efficacy was influenced by acupoints and individual variance. Mechanistically, studies with animal models of FD and patients have shown that acupuncture, a non-invasive strategy for nerve stimulation, may have the potential to control intestinal inflammation and suppress acid-secretion via different somatic autonomic reflex pathways, regulate the brain-gut axis through intestinal microbiota, and has the potential to ameliorate FD-symptoms. The cumulative evidence demonstrated that acupuncture is a promising treatment to alleviate symptoms of FD patients.

## Introduction

Functional dyspepsia (FD), a chronic functional gastrointestinal disorder (FGID), is characterized by upper abdominal symptoms without any organic, systemic, or metabolic diseases (Vanheel and Farré, 2013). Postprandial distress syndrome (PDS) with meal-related symptoms of postprandial fullness, early satiation, epigastric pain syndrome (EPS) with meal-unrelated epigastric pain and burning were proposed by the Rome III consensus and reiterated in the Rome IV revision (Wauters et al., 2020a). The prevalence of FD worldwide has reached 15–20% in 2015 and was markedly increased by 1% annually per year, however, the bulk of FD patients did not seek medical advice (Tack and Talley, 2013). In the United States, FD hardly inevitably increased absence from duty and health care costs in adult individuals (Brook et al., 2010). Despite the familiar occurrence of FD with impressive medical expenses and poor quality of life, the current treatment options are limited due to poorly understood etiopathogenesis (Wauters et al., 2020b).

Acupuncture, in use for the past 3,000 years, is a valuable therapy to improve gastrointestinal symptoms (Zhang et al., 2014). Emphasis was directed at the somatic internal organ connections in the primitive meridian channel theory, indicating that acupuncture stimulation at abdominal and hindlimb regions has been emerging as a potential therapeutic regimen to regulate internal organ function for long distance (Liu et al., 2020) with a low incidence of adverse events (Chen and Michalsen, 2017). Meanwhile, acupuncture improved gastrointestinal dysmotility (Yang et al., 2021) and suppressed visceral pain and acid secretion (Song G. et al., 2019), all of which implied that acupuncture may be conducive for patients with FGID. Concomitant with the increasing use of acupuncture, related mechanisms have been increasingly accumulated and gradually performed. It’s time to summarize the efficacy and potential mechanisms of acupuncture on FD to guide future laboratory and clinical studies.

## Sources and Selection Criteria

We searched PubMed, Web of Science, and Embase. The searches identified English language papers published from the database establishment up to the present time. Keywords included “acupuncture” or “electroacupuncture” or “EA,” and “functional dyspepsia” or “functional gastrointestinal diseases” or “dyspepsia” or “idiopathic dyspepsia” or “non-ulcer dyspepsia.” After being carefully evaluated, the information presented in the following studies was described and discussed.

## Acupuncture

Acupuncture is the use of a needle under the guidance of Traditional Chinese Medicine theory (TCM). Depending on the certain angle the needle pierces the body of the patients, the manipulation of twisting, lifting and thrusting of the acupuncture needle can stimulate a specific part of the body to achieve the purpose of treating disease. The book *Inner Classic of the Yellow Emperor*, the first document that described a complete and laborious theory of the meridians and collaterals, represented a new milestone in the history of acupuncture’s evolution. According to TCM, the meridians and collaterals pertain to the internal organs and extend to the extremities and joints exteriorly, integrating the internal organs, tissues, and other organs into an organic whole, by which they transport *qi*, keeping the functions and activities of all body parts in harmony (Ifrim Chen et al., 2019). In Chinese acceptance, *qi* is vital energy, like the Greek notion of *pneuma* (Kaptchuk, 2002), that flows through these meridians and participates in the homeostatic regulation of the various functions of the body (Kuo et al., 2004; Tian et al., 2014). Most importantly, when the *qi* flow in meridians was impaired or out of balance, organ dysfunction appeared and the associated illness ensued (Sirois, 2014). Acupuncture stimulated the *qi* flow along an involved channel and normalized the *qi* imbalance to harmonize or balance the energy and blood flow through the body (Lee et al., 2008; Tan et al., 2009), which brought the organ back to normality (Ouyang and Chen, 2004). Therefore, *de qi*, or achieving *qi* was the sign of the optimal effect of needle manipulation and was considered as the *sine qua non* of acupuncture for the achievement of a clinically therapeutic effect according to TCM (Tian et al., 2014). The sensations of *de qi* could be perceived by patients with numbness, heaviness, distention, soreness, and spreading sensation, and by acupuncturists as heavy and tight sensation coming from beneath the needle (Song H. S. et al., 2019).

### Methodology of Acupuncture

Based on the method of implementation, acupuncture was mainly classified as acupuncture (manual) and electroacupuncture. In clinical practice, the most commonly used method was manual acupuncture which was performed by an acupuncturist with different maneuvers, such as lifting, thrusting, twisting, and other complex combinations, to achieve *qi* after inserting needles into acupoints. Manipulation played an important factor to induce the needling sensation and bring about the desired therapeutic results. Therefore, acupuncture with manual manipulation was normally used to achieve the expected sensation via local nerve (Chang et al., 2019), blood (Uchida et al., 2019), or neurohumoral system (Zhao, 2008), in turn to achieve optimal performance in individual patients (Chen et al., 2018). However, needling manipulation was difficult to be standardized and popularized in clinical studies because it relied on the experiences and precision of the practitioner with the needle insertion, especially in multicenter studies (Guo et al., 2020).

Electroacupuncture is traditional acupuncture combined with consistent electrical stimulation. While acupuncture induced mechanical stimulation in neuromuscular junctions and caused the local release of neuromodulators, electroacupuncture represented a transdermal electrical stimulation of the nerves with voltage-dependent effects (Kagitani et al., 2010; Ulloa et al., 2017). Therefore, electroacupuncture may be designed to generate nerve excitement and muscle contraction around the inserted needle (Yang et al., 2021) to mimic manual manipulation of acupuncture (Chen et al., 2018). Meanwhile, compared with manual acupuncture, electroacupuncture was more consistent and reproducible, so electroacupuncture was the most commonly used method in clinical trials and laboratory research (Mayor, 2013).

### Identifying Acupoints

**FIGURE 1:**
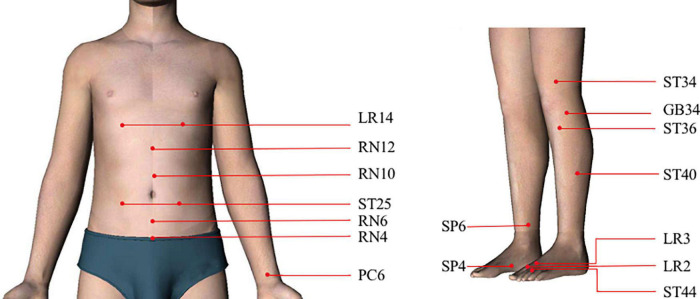
Human maps of acupoints used in functional dyspepsia (FD) studies.

Acupuncture points (acupoints) are special modes on the meridians and enriched with nerve, vascular, and immune cells which connect to the specific organs and modulate the related body functions (Song G. et al., 2019). With respect to acupuncture theory, the selection of different acupoints has a powerful influence on the therapeutic effects of acupuncture including clinical and theoretical research (Yang et al., 2010; Yang N. N. et al., 2020). Modern studies also found a few organizational rules regarding how acupuncture drives distinct somatosensory autonomic pathways, including acupoint selectivity (Ma, 2020). The approaches of how to choose specific acupoints for the treatment of FD were regional and heterotopic points. Regional points were specific gathering points that have an effect on energy redistribution for symptom-specific conditions (Millstine et al., 2017). Therefore, each point located in a particular area was able to treat any disorder of the nearby tissues and organs, which was the common feature of acupoints. For FD, *Tianshu* (ST25), *Qimen* (LR14), *Guanyuan* (RN4), *Qihai* (RN6), *Xiawan* (RN10), and *Zhongwan* (RN12) were commonly used (Hongzhi et al., 2021)and all acupoints were located in the abdominal areas (Figure 1). Remote therapeutic properties were the basic regularity of the points in the meridians. Acupoints located below the elbow and knee joints were effective not only for local disorders but also for *zang-fu* disorders so far as the course of their pertaining meridians could reach (Ifrim Chen et al., 2019). Therefore, heterotopic areas such as points on the upper extremities (*Neiguan*, PC6) and lower extremities (*Xingjian*, LR2; *Taichong*, LR3; *Gongsun*, SP4; *Sanyinjiao*, SP6; *Yanglingquan*, GB34; *Liangqiu*, ST34; *Zusanli*, ST36; *Fenglong*, ST40; and *Neiting*, ST44) were also used to treat FD (Hongzhi et al., 2021; Figure 1).

## Clinical Evidence for Acupuncture on Functional Dyspepsia

The promising efficacy of acupuncture on FD patients has been confirmed by several systemic reviews and meta-analyses. On the basis of current procurable evidence, the significant effects of acupuncture in ameliorating the dyspepsia symptoms were manifested, and even superior to medication (Pang et al., 2016). Meanwhile, the therapeutic function of acupuncture even could sustain 6 months in some clinical studies. Several meta-analyses and systemic reviews related to FD were presented in Table 1 (Lan et al., 2014; Kim et al., 2015; Pang et al., 2016; Zhou et al., 2016; Ho et al., 2017; Guo et al., 2020; Zhang et al., 2020).

**TABLE 1:** Effect of acupuncture on functional dyspepsia.

Year	Journal	Authors	Type of study	Sample size	Statistic value	Conclusion
2020	Evid Based Complement Alternat Med	Zhang et al.	Systemic review	3301	*P* ≤ 0.01	Five types of acupuncture (manual acupuncture, acupoint application, moxibustion, acupoint catgut embedding, and warm acupuncture alone) all were superior to prokinetics and sham acupuncture in terms of improving the symptoms of functional dyspepsia.
2020	World J Gastroenterol	Guo et al.	Systemic review	642	*P* < 0.05	Positive effects of acupuncture and EA were observed in regulating gastric motility, gastric accommodation, mental status, gastrointestinal hormones, and central and autonomic functions while improving dyspeptic symptoms and quality of life.
2017	Sci Rep	Ho et al.	Meta-analysis	1727	*P* ≤ 0.001	Manual acupuncture has marginally stronger effect in alleviating global FD symptoms, compared to domperidone or itopride.
2016	J Altern Complement Med	Zhou et al.	Meta-analysis	3097	*P* < 0.001	Acupuncture appears to be efficacious in relieving FD symptoms and improving quality of life
2016	Evid Based Complement Alternat Med	Pang et al.	Systemic review	1436	*P* ≤ 0.02	Acupuncture therapy achieves statistically significant effect for FD in comparison with sham acupuncture and is superior to medication (prokinetic agents) in improving the symptoms and quality of life of FD patients.
2015	Complement Ther Med	Kim et al.	Systemic review	1423	95% CI 1.85-3.82	Acupuncture treatment was associated with a significant positive effect in patients with functional dyspepsia
2014	Cochrane Database Syst Rev	Lan et al.	Systemic review	542	*P* > 0.05	It remains unknown whether manual acupuncture or electroacupuncture is more effective or safer than other treatments for patients with FD

Several randomized clinical trials (RCTs) show that acupuncture was equally effective as drugs in improving the symptoms of FD including postprandial fullness and early satiation, with no major adverse event (Park et al., 2009; Lima et al., 2013; Ko et al., 2016; Liu et al., 2017; Zheng et al., 2018). In addition, our previous study found that the effects of acupuncture were manifested after the 4 week treatment, and the improvement was maintained during a 12-week post-treatment follow-up (Yang J. W. et al., 2020). In this RCT, 278 PDS patients were randomly divided into an acupuncture group or sham acupuncture group to receive treatment three times per week for 4 weeks. The response rate based on the overall treatment effect and the elimination rate of cardinal symptoms such as postprandial fullness, upper abdominal bloating, and early satiation were the primary outcomes. Compared with sham acupuncture, acupuncture resulted in an increased response rate and elimination rate of all three cardinal symptoms, with sustained efficacy over 12 weeks (Yang J. W. et al., 2020). Similarly, the efficacy of electroacupuncture or sham electroacupuncture was compared in another RCT of 200 patients with refractory FD (Zheng et al., 2018). The results showed relief in the dyspeptic symptoms and more improvements in the scores of Leeds Dyspepsia Questionnaire and Nepean Dyspepsia Index (NDI) in the electroacupuncture group, but not the sham group after 20 treatments in 4 weeks. Meanwhile, the 712 eligible FD patients received acupoint treatment 20 sessions in 4 weeks (Ma et al., 2012). After treatment, the improvement of dyspepsia symptoms and quality of life were found in all groups, which were sustained at 12 weeks. Besides, the overall response rate and quality of life improvement were significantly higher in this group which was stimulated at specific acupoints of the stomach meridian, and lower in the sham acupuncture group, compared with itopride and other acupuncture groups. Therefore, the efficacy of acupuncture in FD patients was well investigated by numerous RCT.

## Proposed Mechanisms of Acupuncture for Functional Dyspepsia

Gastric motor and sensory dysfunction were thought to be the physiological abnormalities that directly cause FD symptoms. Gastric dysmotility including gastric dysaccommodation and delayed gastric emptying occurred in up to 40% patients with FD and was associated with symptoms of postprandial fullness, early satiation, nausea, vomiting, and pain (Tack et al., 2001; Sarnelli et al., 2003; Bisschops and Tack, 2007). Similarly, the prevalence of gastric sensory dysfunction (i.e., hypersensitivity) ranged between 34% and 66% in FD (Rhee et al., 2000; Tack et al., 2001). Targeting gastric motor and sensory dysfunction with acotiamide could antagonize M1 and M2 muscarinic receptors, which triggered the gastric accommodation reflex, increased gastric emptying rate, and ameliorated symptoms in FD patients by inhibiting acetylcholinesterase release (Ogishima et al., 2000; Yamawaki et al., 2018). Acupuncture may have the potential to alter gastric motor and sensory dysfunction, which provided a plausible explanation for the therapeutic effect of acupuncture in some FD patients (Ma et al., 2012; Ko et al., 2016; Zheng et al., 2018).

Traditional FD has been conceptualized as brain-gut disorders, with subgroups of patients demonstrating visceral hypersensitivity and motility abnormalities as well as psychological distress. Although structural or biochemical change was an exclusion criterion in FD, recent studies have shown that there were tangible but subtle disorders of gastroduodenal changes by low-grade inflammation, brain-gut axis, and dysbiosis, which may be amenable to tackle the root cause of disease rather than only alleviate symptoms.

### Targeting Gastroduodenal Low-Grade Inflammation in Functional Dyspepsia

Abnormal mucosal integrity and low-grade immune activation in the gastroduodenal area associated with gastric motor and sensory dysfunction were thought to be the origination of FGID, such as irritable bowel syndrome (IBS), gastroesophageal reflux disease (GERD), and FD (Kaji et al., 2010). In FD studies, it was a reasonable assumption that mucosal barrier dysfunction increased the infiltration of luminal antigens from the enteric cavity and then triggered a low-grade inflammation that generated FD symptoms (Vanheel and Farré, 2013). Low-grade immune activation, especially the expansion of activated eosinophils, potentially resulted from and might also lead to the changing of duodenal permeability.

**FIGURE 2:**
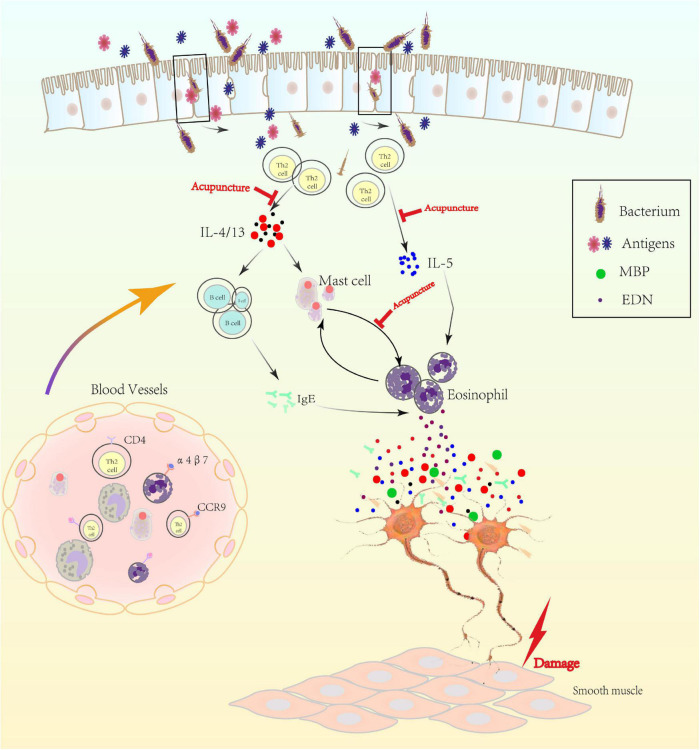
The mechanism of low-grade inflammation in gastroduodenal tissue. Th2 cell was activated in the duodenum, possibly by bacterium or antigens, which crossed the epithelium after impairing of mucosal integrity. Activated eosinophil acted as antigen-presenting cell to Th2-lymphocytes with Ig class switching of B cells to proallergic IgE-antibodies via IL-4 or IL-13. Besides, Th2 cell was the key driver of mast cell and eosinophil via IL-4/13 or IL-5, separately. Activated eosinophil released major basic protein (MBP), eosinophil derived neurotoxin (EDN) and others, which regulated the plasticity of peripheral nerve and then caused visceral hypersensitivity. Gut-homing T cells and other immune cells (expressing α4β7 and CCR9) may also increase in number and produce excess inflammatory cytokines that could increase vascular permeability and alter smooth muscle contraction and then delay gastric empty.

Up to 40% of FD patients have been troubled with duodenal inflammation showing subtle duodenal eosinophilia and degranulation of excess eosinophilia which was adjacent to nerves (Cirillo et al., 2015; Talley and Ford, 2015). Eosinophil was rarely encountered in the blood but more abundant in intestinal muscularis (Griseri et al., 2015). When triggered, eosinophil produced the innate pro-inflammatory cytokines and generated transforming growth factors associated with the polarization of Th1/Th2 (Addula et al., 2018). Meanwhile, Th2 cell was a potent activator of eosinopoiesis and concomitant increasing of activated and tissue-toxic eosinophil in the inflamed intestine, all of which further contributed to duodenal inflammation by releasing factors (Molina-Infante et al., 2014; Cianferoni and Spergel, 2015). Mast cell and eosinophil were the major interesting cells, which were interested in Th2 innate immune responses. Mast cell could also recruit eosinophil in FD, which has been shown to increase in the duodenum (Vanheel et al., 2014, 2018; Wilder-Smith et al., 2019). Collectively, the mechanistic association among these immune cells, epithelium, intestinal microbiota, and various nerves could increase vascular permeability and alter smooth muscle contraction, producing regional intestinal hypersensitivity and motor dysfunction (Figure 2).

IBS and GERD with FD were more than expected by chance, suggesting these disorders may share a common underlying etiopathogenesis-duodenal eosinophilia. Proximal small intestinal eosinophilia may develop FD, not IBS. Reciprocally, those with distal small intestinal or colonic immune activation may produce IBS, while those with more extensive intestinal involvement may develop both IBS and FD (Kaji et al., 2010). Duodenal motor dysfunction by immune activation in FD may, in turn, increase duodenal acid contact time and then induce increased transient lower esophageal sphincter relaxations, a key mechanism of GERD, which may account for the increased risk of GERD symptoms with duodenal eosinophilia (Lee et al., 2004; Ronkainen et al., 2019). Therefore, duodenal eosinophilia, the origination of functional gastrointestinal disorders, has been linked to symptoms of early satiety and pain, barrier disruption and mucosal integrity (Vanheel et al., 2014; Walker et al., 2014).

Since the 1970s, studies have found that acupuncture stimulation at abdominal and hindlimb regions was effective for various immune-related diseases including allergic disorders, infections, inflammatory diseases, and autoimmune diseases, where T cells over-activation or unbalanced Th1/Th2 immune responses played a pivotal role in co-occurred with an induced immune shift (Yu et al., 2014; Wang et al., 2017). Recently, acupuncture was reported to suppress inflammation which was associated with gastroduodenal controls (Liu et al., 2020; Yang et al., 2021). Park et al. (2004) found that electroacupuncture at ST36 led to marked reduction of antigen-specific IgE in serum via suppressing the production of Th2 cytokines, especially IL-4. However, such reduction was prevented by phentolamine, an α-adrenoceptor antagonist, indicating that noradrenergic signaling played a central role in the immunomodulation of electroacupuncture (Yim et al., 2007). Therefore, acupuncture treatment might have an immunomodulatory effect under Th2-skewed conditions to maintain homeostasis (Kim and Bae, 2010).

Acupuncture remarkably suppressed IgE and cytokine production, decreased the number of mast cells, and curbed mast cell degranulation by promoting the cannabinoid CB2 receptor expression (Wang et al., 2019; Zhao et al., 2020). Meanwhile, Yang J. W. et al. (2020) found that electroacupuncture also suppressed pro-inflammatory cytokines releasing in the colonic mast cell via decreasing the toll-like receptor 4 (TLR4) expression, which ameliorated visceral hypersensitivity (Yang et al., 2019). Inhibition of duodenal mast cell degranulation may be one of the potential mechanisms of acupuncture in FD.

The bidirectional communication between the nervous system and immune response allowed the nerve to sense inflammation, activated specific neuronal networks to control immune cells, and then avoided the detrimental effects of excessive inflammation (Ulloa et al., 2017). Acupuncture, as a non-invasive strategy for nerve stimulation, may have the potential to control intestinal inflammation via the somato-autonomic reflex pathway. Luis Ulloa found that electroacupuncture at ST36 controlled systemic inflammation by inducing vagal activation of aromatic L-amino acid decarboxylase, leading to the production of dopamine in the adrenal medulla (Torres-Rosas et al., 2014). Similarly, our previous study also found that electroacupuncture at the hindlimb ST36 acupoint activated the sciatic nerve, which inhibited the expression of the gamma absorptiometry aminobutyric acid (GABA_A_) receptor in the vagal dorsal motor nucleus (DMV) cholinergic neurons to the excited vagal nerve, and in turn controlled local inflammation by suppressing the activation and recruitment of immune cells (Yang et al., 2021). Although electroacupuncture at abdominal acupoints could not activate this vagal reflex to control inflammation, it had the potential to drive spinal sympathetic reflexes to do this, including activation of the splenic sympathetic pathway (Ma, 2020). Ma (2020) showed that 3 mA electroacupuncture at the abdominal ST25 acupoint produced anti-inflammatory effects via driving a spinal-sympathetic axis to activation of β2 adrenergic receptors in splenic cells (Liu et al., 2020). These studies clearly illustrated that driving distinct autonomic pathways from regional acupoints or heterotopic points positively controlled inflammatory response and modulated gastrointestinal function.

Although mild duodenal inflammation, a character with eosinophilic increasing and degranulation, and infiltration of T cells, was capable of giving rise to new ideas into immune-mediated pathophysiology, the mechanisms behind the action of acupuncture on gastroduodenal low-grade inflammation remained enigmatic. Firstly, intestinal eosinophilia, a key role in FD pathology, could be driven by acupuncture from tissue damage to protective role, however, the underlying mechanisms are still unclear. Besides, present studies support the assumption that there was a potential communication between dysfunction of mucosal integrity and low-grade immune activation in the duodenum, but the causal relationship between these two factors in acupuncture needs to be further established. Obviously, there are far more questions than answers in this area. More studies need to be done in these fields, which broaden our understanding of the importance in immune regulation by acupuncture stimulation, and for some gastrointestinal disorders, especially in FD.

### Targeting Brain-Gut Axis in Functional Dyspepsia

The communication between the brain and gut is not a one-way, but a two-way highway, through which the two organ systems exchange under mutual coordination. The pathological repercussions of disordered brain-gut dialog were probably especially pertinent in FD (Powell et al., 2017), which were defined by Rome IV criteria as disorders of brain-gut character with brain processing dysfunction and luminal dysbiosis changing (Tack and Drossman, 2017; Wauters et al., 2020b). There was a reasonable postulation that the brain-gut axis might originate from luminal dysbiosis and ingest constituents interacting with the microbiome and mucosa. Meanwhile, gut microbiota was known to regulate the intestinal barrier and mucosal T cells, and when the balance of gut microbiota was altered, it induced visceral pain responses, intestinal permeability increasing, brain function and behavior dysfunction (Keightley et al., 2015; Holzer et al., 2017).

**FIGURE 3:**
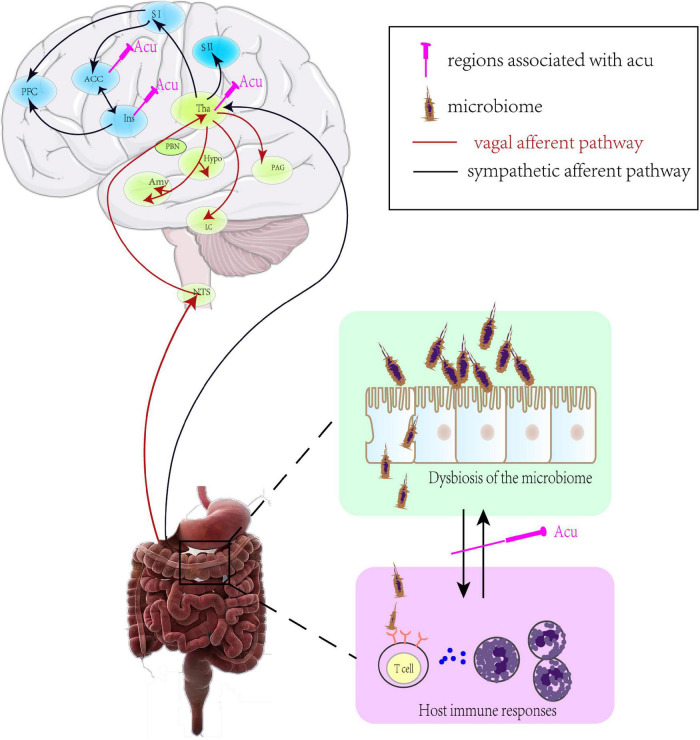
The mechanism of brain-gut axis in FD. ACC, anterior cingulate cortex; PFC, prefrontal cortex; SI(II), primary (secondary) somatosensory cortex; Tha, thalamus; PBN, parabrachial nucleus; Hypo, hypothalamus; PAG, periaqueductal gray; Amy, amygdala; LC, locus coeruleus; NTS, nucleus of the solitary tract; Acu, acupuncture.

Healthy human gastrointestinal microbiology analysis found that there were two main phyla (*Firmicutes* and *Bacteroidetes*) that accounted for more than 90% of microbial communities, while *Actinobacteria* and *Proteobacteria* were minor contributions. The intestine-microbiome played a key role in the pathogenesis of numerous gastrointestinal diseases, including FD. Growing evidence indicated that the changes of microbiota were not apparent phenomena secondary to disease, but instead played an indispensable role in the pathogenesis and clinical process of the disease. Besides, intestinal microbiota had capacity to change brain function by altering the balance of host immune responses, particularly in the differentiation of T cells (Powell et al., 2012; Furusawa et al., 2013; Atarashi et al., 2015; Seo et al., 2015). When microbiota and immune response were altered, the visceral sensory signals from the gut to the brain were conveyed through vagal or sympathetic afferent. Vagal primary afferent carried the visceral sensations to the hypothalamus, locus coeruleus (LC)-amygdala system, and periaqueductal gray (PAG) via the nucleus of the solitary tract (NTS) (Van Oudenhove et al., 2004; Dunckley et al., 2005), while the sympathetic nerve carried them to the somatosensory cortices (SI/SII), cingulate cortex and the insula via spinothalamic tract traveling (Almeida et al., 2004; Van Oudenhove et al., 2004; Jones et al., 2006; Figure 3).

Although dysfunction of the gut-brain axis was driven by flora imbalance, it was also quite obvious that central nervous dysfunction in FD was bidirectional. It has been demonstrated that psychosocial stress was responsible for aggravating FGID severity via disturbing brain function in the gastrointestinal inflammatory model (Reber, 2012; Holzer et al., 2017). An ocean of changes was gradually appearing in the hypothalamic-pituitary-adrenal (HPA) axis and its adrenocortical hormonal system, autonomic nervous system, and immune response under stress. The HPA axis appeared to be the most important responder to stress (Goldstein and Kopin, 2008) with corticotropin releasing factor (CRF) as a mediator operating both in the brain and gastrointestinal tract (Taché and Million, 2015). Compared with healthy characters, higher levels of glycometabolism in the insula, anterior cingulate cortex (ACC), cerebellum, thalamus, and middle cingulate cortex (MCC) were found in FD patients, which positively correlated with the symptom index of dyspepsia (SID) score and NDI score (Zeng et al., 2011).

Due to the development of neuroimaging techniques like functional Magnetic Resonance Imaging (fMRI), increasing numbers of studies have presented that the central nervous system was indispensable in acupuncture treatment (Han et al., 1982; Takahashi, 2011). A neuroimaging study found that acupuncture stimulation suppressed the level of glycometabolism in the insula, thalamus, brainstem, ACC, and hypothalamus, which were positively correlated with the SID score and negatively related with the NDI score in FD patients (Zeng et al., 2012).Also, recent clinical research has found that different acupoint stimulation activated relatively different brain regions but with similar clinical efficacy, indicating that different acupoints might both activate sensory transduction regions, such as the brainstem, thalamus, and visceral modulation regions for similar effects (Zeng et al., 2015). In IBS rats, electroacupuncture relieved mood disorder and repaired the intestinal mucosal barrier by decreasing hypothalamic and gastrointestinal expression of CRF, indicating a potentially dual therapeutic role for acupuncture in regulating disorders of gut-brain interaction in the FGID model (Chen et al., 2019). Meanwhile, acupuncture could adjust the count and proportion of the intestinal microbiota to recover its stability and ameliorate the intestinal barrier function by promoting the interaction between intestinal microbiota and brain-gut axis, and then suppress the production of proinflammatory cytokines (Figure 3). Alpha and beta diversity were significantly decreased in FGID model animals, which was shifted toward the phyla of *Bacteroidetes, Firmicutes, Fusobacteria*, and *Proteobacteria*. Electroacupuncture at ST25 and ST36 alleviated symptoms of visceral hypersensitivity by decreasing in the phyla of *Fusobacteria* and then downregulating the levels of pro-inflammatory factor IL-18 in colon tissue in the IBS model (Song et al., 2020). Neurobiological processes were modified by the bi-directional communication that occurred along the gut-brain axis, so an imbalance of the gut-brain axis by gut microbial dysbiosis may also play important role in neurodegenerative disease (Felice et al., 2016). In the model of Parkinson’s disease, acupuncture improved motor function and comorbidity by regulating the gut microbial dysbiosis and thus inhibiting the neuroinflammatory responses and apoptosis in both the striatum and the substantia nigra (Jang et al., 2020). These results indicate that the brain-gut axis may be a promising target for acupuncture treatment in FD patients.

The role of immune response was considered as the gatekeeper and master regulator of the brain-gut axis. Therefore, brain-gut axis dysfunction rooting from gut microbial dysbiosis increased the production of pro-inflammatory cytokines and epithelial permeability, which further aggravated immune response in the duodenum. Here, growing evidence indicated that the intestinal microbiome was capable of change through acupuncture, it could be a promising therapeutic target in acupoint stimulation. Therefore, we proposed an assumption that acupuncture may act on the following two pathways to suppress the inflammation and ameliorate FD symptoms: (1) inhibition of the releasing inflammatory cytokines via different somatic autonomic reflex pathways; and (2) regulation of the brain-gut axis through intestinal microbiota. However, it should be pointed that the gut microbiota, immune cells, autonomic nerve, and brain regions were interactive and function in a complex circuit system and by unclear mechanisms. Therefore, difficulties in studying and comprehending the vast range and interactions of microbiota presently preclude any comprehensive understanding of acupuncture in FD.

### Targeting Duodenal Acid in Functional Dyspepsia

Although the pathogenesis of the barrier defect and immune activation in FD was still inconclusive, duodenal acid, lipids, stress, and other components were likely candidates. Duodenal acid perfusion resulted in mucosal hyperpermeability and immune cell activation that has been implicated in delaying gastric emptying (Schwartz et al., 2001a,b), and impairing accommodation and hypersensitivity to distension in healthy individuals (Simrén et al., 2003; Lee et al., 2006; Vanuytsel et al., 2011). However, gastric acid secretion was normal in some FD patients, indicating that duodenal acid exposed possibly due to duodenal hypersensitivity to acid (Schwartz et al., 2001b) or low clearance of duodenal acid, rather than increasing of acid secretion (Samsom et al., 1999; Schwartz et al., 2001a). A clinical study by endoscopists demonstrated that, compared with healthy volunteers, FD patients were hypersensitive to acid, which was related to more severe dyspeptic symptoms (Ishii et al., 2010). By contrast, several studies indicated that duodenal acidity did not correlate with the severity of dyspeptic symptoms (Lee et al., 2004; Bratten and Jones, 2009). Therefore, these results indicated that duodenal acid exposure did not directly contribute to the development of symptoms, it might impair gastric motor and sensory function, and then activate low-grade immune to symptom generation.

Since the 1970s, acupuncture with various regimens given for 6 weeks was reported to reduce gastric acid secretion in patients with dyspepsia (Sodipo and Falaiye, 1979; Lux et al., 1994). Compared with placebo acupuncture, acupuncture significantly reduced the level of basal and maximal acid output, which could be reversed by naloxone, suggesting that the antisecretory effect of acupuncture was mediated by opioid pathways (Sodipo and Falaiye, 1979; Tougas et al., 1992; Lux et al., 1994). Meanwhile, in conscious dogs, acupuncture also decreased gastric secretion of acid, which was completely blocked by the local anesthetic agent or anticholinergic agent, indicating that the effect of acupuncture in acid secretion was associated with somatic-visceral reflex mechanism (Zhou and Chey, 1984).

The studies of acupuncture in acid secretion have become obsolete, most of which were performance before Roma III, so another possibility that cannot be ruled out was that acupuncture treatment in the decreased acid secretion of the duodenum may be a consequence of other abnormalities especially in hypersensitivity. Future studies are needed to explore the abnormality of duodenal acid and to define its role in symptom amelioration by acupuncture in FD.

## Discussion

### Acupuncture vs. Pharmacological Treatments

Current guidelines and expert consensus recommended the use of prokinetics and acid-suppressive drugs as one of the routine treatments for PDS and EPS to amelioration early satiation or postprandial fullness and epigastric pain or burning, respectively (Ford et al., 2020). However, the effectiveness of pharmacological treatments remained unsatisfactory with 10-week treatment and potential side effects of medicines raised concern on their longer use (Quigley, 2017; Ou et al., 2021). Acupuncture, as a promising non-pharmacological treatment, could provide relief from symptoms in FD patients with mild or intermittent symptoms by targeting low-grade inflammation, brain-gut axis, dysbiosis, or acid-secreting. A systematic review and meta-analysis with 16 RCTs involving 1,436 participants found that acupuncture therapy was superior to prokinetic agents in improving the symptoms and quality of life in FD patients (Pang et al., 2016). Meanwhile, some special populations, including the elderly and children, prefer non-pharmacological options in FD because pharmacological treatment also came with considerable risks for harm, such as the increased risk of extrapyramidal reactions, sudden cardiac death, and drug-induced neurological disorders (Ho et al., 2017). Therefore, compared with pharmacological treatments, acupoint stimulation was at least as effective as or possibly more suitable, with a very rare occurrence of side effects for mild FD or special patients.

Acupuncture may be an adjunct treatment in severe or refractory FD. Accumulating evidence had demonstrated that acupuncture combined with medication was more effective than medication alone. The term “dyspepsia” indicated a constellation of symptoms with different underlying mechanisms, and no single drug could reasonably be expected to treat them all, which may be the main reason for unsatisfactory drug effectiveness in FD. Pharmacological treatment combined with acupuncture as a multi-target therapy, may be a promising avenue for severe FD. An overview of systematic reviews and network meta-analysis found that compared with acupuncture or drugs alone, the combination of manual acupuncture and clebopride was the most effective treatment in alleviating FD symptoms (Ho et al., 2017). Meanwhile, acupuncture may be used in patients with refractory FD who had poorer responses to conventional medical therapy or suffered from serious side effects. An RCT with 287 patients of refractory FD found that compared with sham electroacupuncture, electroacupuncture efficaciously improved dyspeptic symptoms after a 4-week treatment with sustained efficacy over 20 weeks (Zheng et al., 2018). Similarly, a pragmatic randomized trial with 132 participants also found that electroacupuncture plus on-demand gastrocaine provided significant, clinically relevant symptom relief compared to on-demand gastrocaine alone (Chung et al., 2019). Acupuncture combined with pharmacological treatment may have the potential to reduce the side effects of medical treatment, but it remained controversial due to limited research.

In summary, acupuncture could relieve mild or intermittent symptoms of FD, and was helpful in the special patients both as an isolated and adjunct treatment. It was emphasized that acupuncture combined with pharmacological treatment not only enhanced the improvement FD symptoms but also reduced the side effects of the medical treatment which were the main cause for high dropout rates with drug treatment.

### Influence Factors of Acupuncture

Acupuncture, the stimulation of specific acupoints on the body with needles, was gradually accepted as part of their current healthcare regimen in patients, especially in FGID patients. An important feature of acupuncture was that the chosen acupoints and the individual variance could affect the responsiveness of acupuncture.

#### Types of Functional Dyspepsia

Based on the dyspepsia symptoms, FD may be grouped into PDS and EPS. Although the need for different therapeutics approaches in treating EPS and PDS remained controversial, acupuncture produced different therapeutic responses in two types of FD patients. A retrospective analysis of an RCT (Ma et al., 2015) found that PDS patients in the acupuncture group showed higher response rate and more score-changes of postprandial fullness, early satiation, and quality of life compared to sham acupuncture and itopride group, but not in EPS, demonstrating that acupuncture was effective against meal-related FD symptoms only. Similarly, Park et al. (2009) reported that there was no significant difference between acupuncture and sham acupuncture in alleviating epigastric pain and epigastric burning. The distinct therapeutic effects of acupuncture on PDS and EPS may be explained by the following two reasons: (1) Acupuncture promoted the gastrointestinal motility via the different somatic autonomic reflex pathways, which has been shown to correlate with postprandial fullness and early satiation in PDS (Kusano et al., 2011; Ulloa et al., 2017; Yang et al., 2021). (2) Inflammation in FD, characterized by the increasing of duodenal mucosal eosinophil and mast cell, has been implicated in the underlying cause of the disease process. Some researchers hold the view that the increased eosinophil and mast cell were linked to PDS rather than EPS in most studies and may be presented in more than 50% of those affected by PDS (Talley and Ford, 2015; Talley, 2020). These results indicated that low-grade immune activation in the gastroduodenal area was more related to gastric motor and sensory dysfunction in FD. Therefore, the anti-inflammatory effect of acupuncture may be another reason for the notably therapeutic effects in PDS.

#### Acupoints

Based on traditional meridian and acupoint theories, the efficacy of acupuncture was mostly determined by acupoint. There was a specific effect of acupoint compared with non-acupoint, and the specific effect may differ from acupoints on different meridians or of different types (Ma et al., 2012). A large RCT with 712 FD patients who were assigned to five different acupuncture groups and itopride group, wanted to explore acupoint specificity in different aspects. This research found that the overall response rate and quality of life were significantly higher in acupuncture at specific acupoints of the stomach meridian, and lower in the sham acupuncture group, compared with itopride and other acupuncture groups, indicating that the benefits of acupuncture in FD relied on acupoint specificity (Ma et al., 2012). Therefore, the stomach meridian was the most popular meridian in FD, because it directly connected the gastrointestinal tract and brain. In this meridian, ST25 at abdominal regions and ST34, ST36, ST40, and ST44 at hindlimb regions were commonly used points to promote recovery of gastrointestinal function. It is a logical hypothesis that the underlying mechanisms of acupuncture at regional or heterotopic points on FD were different. The stimulation of homotopic acupoints had an advantage in directly altering gastroduodenal low-grade inflammation and the balance of the microbiome. By contrast, acupuncture at distal regions activated nerve trunk, such as sciatic and median nerves as well as their branches, which activated associated brain regions via neural circuit in turn to regulate the brain-gut axis, and then relieved dyspepsia symptoms in FD. Therefore, acupuncture combined with regional and heterotopic points may be more powerful in improving FD symptoms as arising from different mechanisms.

### A Revisit to Sham Acupuncture

Acupuncture treatment contains the “specific” and the “unspecific” or “placebo” effects. If the intervention was a drug, the “specific” component was the pharmacologically active agent while the placebo was an substance (Dincer and Linde, 2003). However, it became more complicated to distinguish the “specific” or “placebo” effects in acupuncture which was defined as a complex physical intervention. Therefore, clinical studies often faced a difficult situation: verum and sham acupuncture stimulation produced similar therapeutic effects (Moffet, 2009). From the neurobiological point of view, there was no real sham control.

There were 3 commonly used sham controls: (1) superficial needling of the true acupoints; (2) needling of the true acupoints which were not indicated for the condition being treated; and (3) needling of the non-acupoints (outside true acupoints). The various types of sham acupuncture had different advantages and drawbacks. Firstly, depth of needling was a potential modifier of acupuncture effects, and acupuncture at true acupoints superficially was a good test for the influence of the depth of needling. However, there was a dense nerve network within a large group of mechanically sensitive sensory neurons in “true” acupoints, such as mechanically sensitive, unmyelinated C-fiber polymodal nociceptors that respond to light punctate noxious mechanical stimuli (Ma, 2020). In humans, skin pinching stimulation had the potential to activate C-fiber nociceptors and other sensory afferents, and in turn produce pleasant tactile perception which contributed to analgesic effects or produced antipain effects (Cavanaugh et al., 2009). Therefore, superficial needling of the true acupoint not only contained “placebo” effects but also had some “specific” effects of acupoint. Secondly, acupuncture at disease-unrelated acupoints could mimic the manipulation of “true” acupuncture, therefore, it was a good way to clear the acupoint specificity and to blind patients. Ma et al. found that the therapeutic effects of needling acupoints at the stomach meridian were significantly higher than acupoints at the gallbladder meridian in FD patients, which provided evidence for the existence of specificity between acupoints on different meridians (Ma et al., 2012). However, this “sham acupuncture” control was difficult to produce a significant difference between groups. Finally, the correct location of points was vital for acupuncture to hold some validity, so needling at incorrect sites may be a control intervention. Furthermore, some evidence found that the size of differences between groups was related to the location of incorrect points, as differences between experimental and control groups have been larger in trials in which more distant sham points were used (Sánchez Aranjo, 1998; Dincer and Linde, 2003). Due to the spatial and segmental innervation of the somatic acupoints, however, we could not be sure whether this affected the results of the trial, when control acupuncture was performed at close non-acupoints. Although needling at distant non-acupoint was a good way to determine the therapeutic effect of acupuncture, patients could tell true from sham acupuncture by this way.

In conclusion, sham acupuncture could be defined as a useful “inactive” control, so even if acupuncture treatment was not superior to sham acupuncture in some researches, we could not ignore the effect of acupuncture (Lin et al., 2020). Meanwhile, there was no single adequate sham intervention for acupuncture trials and the choice of sham acupuncture should depend on the aims (Dincer and Linde, 2003), such as answering specific questions on the composite influence of the various manipulated variables (location, depth, manipulation, de qi, etc.).

## Summary and Future Directions

Here, we argued that the primary cause of FD was gastroduodenal low-grade inflammation and acid exposure, which impaired mucosal integrity, caused brain-gut axis dysfunction, and impaired brain network connectivity, all of which generated various symptom patterns. Overall the clinical studies indicated that acupuncture was a promising treatment to alleviate symptoms in FD patients, whose efficacy was influenced by the chosen acupoints and the individual variance. Mechanistically, studies with animal models of FD and patients have shown that acupuncture, as a non-invasive strategy for nerve stimulation, may have the potential to control intestinal inflammation and suppress acid-secretion by inhibiting the releasing of inflammatory cytokines by different somatic autonomic reflex pathways and regulation of the brain-gut axis through intestinal microbiota and so has the potential to ameliorate FD-symptoms. Progress in the comprehension of the different underlying pathophysiological mechanisms of acupuncture might lead to better symptom control through the development of therapy, so many challenges and knowledge gaps remain to be studied in this field.

In the future, large and well-designed clinical trials with more reasonable sham interventions are needed to investigate the effect of acupuncture on FD patients. Additionally, female predominance, smoking, emotional disorder, atopic diseases, and autoimmune diseases were the risk factors of FD, however, the influences in acupuncture-treatment response by these factors still need to be identified. Finally, FD, IBS and GERD shared a common underlying etiopathogenesis (Aro et al., 2015; Talley, 2020). Acupuncture may have the potential to improve the comorbidity of these FGIDs by suppressing duodenal immune activation through multi-pathway, but it is waiting to be identified.

## Author Contributions

N-NY, J-WY, and C-ZL put forward the idea of performing the review. N-NY wrote the initial manuscript. J-WY and C-ZL revised and edited the manuscript. C-XT draw the manuscript. Y-JL, L-LL, L-YQ, and YW summarized the tables. All authors have approved the submitted version.

## Conflict of Interest

The authors declare that the research was conducted in the absence of any commercial or financial relationships that could be construed as a potential conflict of interest.

## Publisher’s Note

All claims expressed in this article are solely those of the authors and do not necessarily represent those of their affiliated organizations, or those of the publisher, the editors and the reviewers. Any product that may be evaluated in this article, or claim that may be made by its manufacturer, is not guaranteed or endorsed by the publisher.
